# Supplementary material for: Are carbon emissions trading and green financial instruments synergistic? -Comprehensive quantitative research based on content analysis
Source: PLoS One. 2024 Mar 7;19(3):e0298601. doi: 10.1371/journal.pone.0298601 (PMC10919601; doi:10.1371/journal.pone.0298601)
Supplement: S1 Data — (ZIP) [file pone.0298601.s001.zip › Data/地级市Z_F工作报告.zip!/║╙─╧/╔╠╟≡╩╨/└┤╫╘╣1⁄2╓┌║┼í╛┬φ┐╦╩2╛▌═°í┐_2010.docx]

各位代表：

现在，我代表市人民政府向大会报告工作，请予审议，并请各位政协委员和其他列席人员提出意见。

一、2009年工作回顾

2009年，是新世纪以来我市经济社会发展遇到困难最多、挑战最大的一年。受国际金融危机影响，年初我市经济增速大幅下滑，企业开工严重不足，财税收入增速锐减，前2个月，规模以上企业亏损额同比增加5.7倍，工业增加值、财政一般预算收入增速分别回落19和35.6个百分点。沿海企业用工需求严重不足，近100万农民工集中返乡，劳务收入大幅减少，就业形势严峻。面对异常困难局面，市政府在省委、省政府和市委的直接领导下，在市人大、市政协的监督支持下，团结带领全市人民，以深入学习实践科学发展观为动力，以保增长、保民生、保稳定为主线，坚决贯彻落实战危机、保增长的政策措施，努力克服国际金融危机和特大自然灾害的不利影响，加快经济发展，着力改善民生，促进社会和谐，做出了决战二季度、大干三季度、决胜四季度的总体部署，坚定信心，迎难而上，齐心协力，克难攻坚，较好地完成了市三届人大四次会议确定的各项目标任务，保持了经济社会发展的良好势头。

(一)经济总量质量迈上新台阶。全年生产总值首次突破千亿元大关，达1032.9亿元，增长11.2%，高于全省平均水平。三次产业结构比例为26.2∶44.7∶29.1，第三产业增加值占GDP比重比上年提高1.1个百分点。地方财政总收入70.5亿元，增长13.3%，其中，税收收入占财政一般预算收入的76.6%，居全省第4位;地方财政一般预算收入35.4亿元，增长12%，支出完成151.1亿元，增长28.1%。粮食生产在大灾之年获得丰收，总产达122亿斤，创历史最高纪录。加快产业集聚区建设步伐，先后制定出台了一系列加快产业集聚区科学发展的政策措施，全市11个产业集聚区总体发展规划全部通过省级评审，累计完成基础设施建设投资33.8亿元，产业集聚区内建成投产企业449家，在建企业166家，产业集聚效应初步显现。加强现代产业体系建设，按照“大项目～产业链～产业集群～产业基地”的要求，编制产业发展规划，扩大产业规模，拉长产业链条，提升产业水平和产品竞争力，促进了工业结构优化升级，规模以上工业企业实现利润80.4亿元，增长10%。加快发展商贸物流和信息服务业，完成全社会消费品零售总额343.4亿元，增长19.3%，商丘农产品中心批发市场交易额120.7亿元，位居全国同类市场前列，全球最大的商业零售企业沃尔玛落户商丘，完成3G投资5.53亿元。节能减排和生态建设成效明显，积极淘汰落后产能，关停15.8万千瓦小火电机组，污染减排超额完成年度任务，化学需氧量提前一年实现“十一五”减排目标。环境质量稳步提高，空气环境优良天数338天，位居全省前列。全年植树造林29.3万亩，完成比例居全省第一位。

(二)调控服务能力有了新增强。市政府坚持把保持经济平稳较快发展作为首要任务，围绕搞服务、保企业、扩内需、保增长等关键环节，全面加强对经济工作的领导，切实做好经济运行调节调控工作，按照困难企业保生存、一般企业渡难关、优势企业上水平的工作目标，启动实施“企业服务年”活动，对787家企业实行一对一、一盯一贴身服务。针对年初全市工业经济大幅下滑的严峻形势，从帮助企业解决资金困难、支持企业开拓市场、促进企业强化管理、完善中小企业服务体系等方面入手，及时出台支持工业经济发展的优惠政策，落实铝工业收储贴息、财政补助支持政策，促进了重点区域、重点行业、重点企业的产能恢复，有效遏制了工业经济下滑趋势，规模以上工业增加值增速从3月份的1.4%回升至12月份的30.6%。较早解决我市房地产发展过程中存在的问题，在最短时间内以较快的速度出台了促进二手房交易的政策措施，全年房地产成交面积416万平方米，其中二手房成交82.8万平方米，增长45.2%，引导房地产市场健康发展。大力推进银企合作，金融机构全年新增贷款108.98亿元，超额完成省政府下达我市80亿元的目标任务，居全省第四位，为企业发展提供了资金保障。加大土地资源的供应和配置，全年上报审批土地2.33万亩，比上年增加1325亩。加强煤电油气运调节，保证了重点项目建设和人民群众生产生活的需要。这些调节措施切实改善了企业的生产经营环境，在极其困难的条件下成功遏制了经济下滑，实现了“V”型逆转，使全市经济运行保持了企稳回升、前低后高、持续向好的发展态势。

(三)重点项目建设实现新突破。实施投资拉动战略，开展“项目建设年”活动，实行重大项目联审联批，完善重点项目协调推进机制，组织开展重点项目观摩点评活动，争取中央新增投资28.18亿元，完成重点项目投资258亿元，增长33.6%，超额完成投资任务。神火集团薛湖煤矿、神火集团年产15万吨铝板带箔、泰美车业10万辆电动车配件、七彩纺织、众品食业等106个重点项目竣工，一批重大产业升级和基础设施项目相继落地;中石油商丘千万吨炼油厂项目上报国家，申请列入“十二五”发展规划，商丘机场项目可研报告上报国务院、中央军委审批，国电民权电厂二期、邢商永地方铁路等项目前期工作加快;河南煤化集团20万吨乙二醇项目和千吨级碳纤维项目、供电公司庄周500KV输变电站二期扩容工程和永城500KV输变电站工程、神火集团1�60万千瓦发电机组、郑煤集团商丘中亚化工醇醚项目和商周高速二期、连霍高速改扩建工程相继开工，为今后的持续发展备足了后劲，积蓄了力量。

(四)城乡基本建设呈现新面貌。全社会完成固定资产投资693.17亿元，增长29.9%。加强城区专项规划和详细性规划编制，启动了城乡一体化规划编制，全市控制性详细规划覆盖率42%，城乡规划管理水平不断提高。以创建国家园林城市为契机，加大城市基础设施建设力度，基本完成阏伯路改扩建工程，对神火大道等4条道路部分路段实施了人行道升级改造和中修罩面，长江路Ⅰ桥、Ⅱ桥建成通车，打通了平原北路，完成了睢阳北路道路排水、胜利路大修改造。筹措资金8000万元，组织实施了城区94条道路和背街小巷道路整修改造工作，改善了市民生活环境，方便了百姓出行。污水处理厂二期建设厂区主体工程基本完成，路灯、供热、供气、供水、弱电管网等市政基础设施建设进一步加强。加大对违法用地和违法建设的查处力度，提高了依法用地意识和城市管理水平。整合交通、卫生、教育、扶贫等涉农资金2.63亿元，扎实推进新农村建设，涌现出了一批先进示范村。投资7.1亿元，新建、修复一批农田水利基本设施，增强了抗御自然灾害能力。新建农村公路1000多公里，改造危桥76座，农村生产生活条件不断改善。投资2.24亿元，解决了44.8万农村人口的饮水安全问题。农业机械化水平不断提高，公共气象服务体系逐步完善，防灾减灾能力不断增强。全市城镇化率35.5%，比上年提高2个百分点。

(五)和谐商丘建设取得新成果。集中力量办成了一些多年想办的大事，省定十大实事和市定十件实事总体完成情况良好。城镇居民人均可支配收入12715元，增长8.2%;农民人均纯收入4054元，增长8.1%。积极扩大就业，城镇新增就业9.4万人，下岗失业人员再就业3.6万人，分别完成年度目标任务的168%和174%。帮助97万返乡农民工再就业，实现劳务输出222万人次，创劳务收入152亿元。优先发展教育事业，落实资金9亿元，用于保障和提高农村中小学生均公用经费标准;协调资金4000万元建设市职教中心。加快发展旅游业，游客接待量和旅游收入稳步增长。投入资金16.5亿元，用于发放养老金、城乡困难居民救助、优抚对象和五保供养对象生活补助。新建、收购廉租住房36万平方米，改善了城市低收入家庭的住房状况。虞城县、梁园区被纳入国家第一批新型农村社会养老保险试点，两县(区)60周岁以上农民已领到第一批养老金。全面推行新型农村合作医疗，不断扩大城镇职工、居民医疗保险参保范围，在全省率先把大学生纳入城镇居民基本医疗保险范围，农民参合率91%，城镇职工参保率90%，城镇居民参保率85%。建立手足口病和甲型H1N1流感四级防控体系，确保了人民群众的健康安全。加大支农惠农力度，销售家电、汽车、摩托车等43.6万台，30多万农户受益，落实粮食直补等各项补贴资金11.8亿元。圆满完成灾后倒房重建工作，2.86万户灾民在入冬前全部入住新居。完善计划生育利益导向机制，兑现各种计生优待政策资金8099.76万元，全市人口出生率控制在10.1‰左右。开展食品药品安全专项整治，产品质量和食品药品安全保障水平不断提高。健全完善安全生产工作责任制，全市没有发生重特大安全事故。民族团结、宗教和谐局面进一步巩固。深入推进平安商丘建设，积极开展“信访积案化解年”活动，一大批突出问题得到有效解决，信访工作受到省委、省政府表彰。

(六)改革开放迈出新步伐。市、县两级政府机构改革扎实推进。国企改革和战略重组效果明显，神火集团并购商电铝业集团鑫丰铝厂，壮大了电解铝主业规模，拉长了产业链条，提高了企业效益。南京雨润集团与福源集团、海泰集团与冰熊冷藏车厂、中国建材集团与振华玻璃厂合作重组进一步深化。文化体制改革继续推进，商丘演艺集团被评为全国文化系统先进集体和第三批省文化产业示范基地。医药卫生体制改革稳步推进。市城信社成功改制为商业银行，梁园区、睢阳区农村信用社组建华商农村商业银行获得批准，平安保险、民生证券先后落户商丘。“永煤工程技术中心”被认定为国家级工程技术中心，新增2家省级工程技术中心，2家企业被认定为高新技术企业，7项科技成果获河南省科技进步奖，2个农作物新品种通过国家审定，自主创新体系建设得到加强。认真开展大招商活动，先后组团到长江三角洲、珠江三角洲、台湾等地开展一系列招商和经贸交流活动，成功举办了面品博览会、木兰文化节，加强与民间社团组织的联络，建立重要资讯、重点项目、重大事项定期沟通和协调落实机制，招商引资工作取得新成效，各县(市、区)共引进10个固定资产投资3亿元以上工业项目。加大优化发展环境工作力度，创新“两转两提”工作载体，在全省率先建成并使用了行政效能电子监察系统，压缩了审批时限，提高了办结率。通过市长热线和市长信箱受理人民群众反映的各类问题和建议2.08万件，办结率、反馈率、满意率不断提高，区域经济发展环境综合考评工作在全省名列前茅。

民主法制建设进一步加强，自觉接受人大及其常务委员会的法律监督和人民政协的民主监督，拓宽同人民群众的联系渠道，提高了决策的民主化和科学化。加强政府廉政建设，反腐倡廉工作取得新成效。加强精神文明建设，公民道德水平和城乡文明程度不断提升。外事、侨务、对台工作进一步加强，审计、邮政、盐业、烟草、史志、体育、人民防空、档案管理、社科研究、青少年、妇女儿童、老龄、残疾人和慈善事业等工作都取得了新的成绩。四川地震灾区援建任务全面完成。

各位代表，回顾一年的发展历程，我们真切地感到，困难前所未有，成绩来之不易。一年来战危机、保增长，抗灾害、夺丰收，惠民生、促和谐的实践，充分展示了商丘人民坚韧不拔、自强不息的精神风貌，充分展示了商丘广大干部攻坚克难、奋发有为的能力素质，充分展示了各级各部门团结协作、同舟共济的凝聚力和战斗力!我们不会忘记，在严重的国际金融危机面前，大家以特殊之举应对困难之局，变压力为动力，变挑战为机遇，积极应对，果断出击，危中求机，化危为机，打赢了战危机保增长的总体攻坚战!我们不会忘记，在历史罕见的冬春连旱和“6.3”强飑线特大自然灾害面前，应急响应机制立即启动，全市上下紧急动员，驻豫部队、民兵预备役、武警消防官兵和公安民警冲锋在前，社会各界积极参与，奏响了一曲“万众一心、众志成城、顽强拼搏、共克时艰”的时代凯歌，夺取了抗灾救灾的全面胜利!我们不会忘记，在关系商丘全局的大事面前，全市各级各部门顾全大局，团结协作，主动承担责任，努力克服困难，全力保障支持，2009年我市获得“国家园林城市”、“全国绿化模范城市免检市”、“全国科技进步先进市”、“全国粮食生产先进市”、“全国民族团结进步模范集体”、“中国最具发展潜力城市”、“中国十大和谐民生城市”、“中国特色魅力城市200强”等荣誉称号的背后，就倾注着各驻商单位、各级各部门和广大干部群众的勤劳与智慧、心血和汗水。在此，我代表市人民政府，向全市人民，向人大代表、政协委员，向各民主党派、工商联、各人民团体和社会各界人士，向驻商解放军、武警消防官兵、公安民警和离退休老同志，向参与商丘建设的各位客商和所有关心支持商丘发展的海内外朋友，表示衷心的感谢!致以崇高的敬意!　　　我们也清醒地看到，我市经济社会发展还存在不少亟待解决的困难和问题。一是外部经济环境不确定、不稳定因素很多，在经济复苏缓慢复杂的大背景下，保持我市经济的平稳较快增长困难较大。二是产业结构调整优化任务十分艰巨，我市资源加工型企业居多，企业对资源的依存度过高，产业层次较低，特别是在保持经济平稳较快增长的前提下，着力促进经济转型升级的难度很大，固定资产投资增幅回落等问题不容忽视。三是统筹城乡发展难度较大，基础设施、环保设施、规划建设管理、公共服务体系建设欠账较多，农村人口多，比重大，破解城乡二元结构、统筹城乡协调发展需要付出巨大努力。四是金融服务体系和信用机制还不健全，融资难仍是制约我市经济发展的重要瓶颈。五是一些群众关心的热点难点问题还没有得到有效解决，财政收支矛盾突出，资源和环境的压力较大，维护社会和谐稳定任务繁重。六是政府工作标准、政务管理、服务水平离形势发展的要求和人民群众的期望还有较大距离，一些公务员的精神状态、工作作风、综合素质还有待于进一步提高。对这些问题，我们必须高度重视，认真采取措施，切实加以解决。

二、2010年工作的总体要求和奋斗目标

今年是实施“十一五”规划的冲刺之年，也是巩固经济回升向好势头、为“十二五”规划启动实施奠定基础的关键之年。从总体上看，今年经济发展环境将好于去年。世界经济正在复苏，我国经济回升向好，河南呈现出较好的发展趋势、态势和气势，随着促进中部崛起规划的全面实施和商丘融入中原城市群步伐的加快，我市的区位、市场、成本、要素保障等优势逐渐扩大，加快发展面临着难得的机遇。同时，商丘的发展也充满了压力。我市经济回升的基础仍不牢固，保持经济平稳发展面临较大困难;产业结构向高端化演进、经济结构向低碳化发展的现实要求，使我们的发展模式面临严峻考验;既要保持较快的发展速度，又要转方式、调结构、促转型，还要统筹城乡和经济社会的协调发展，使我们的工作面临“两难”选择。面对组织的重托和人民的期望，政府深感责任重大、使命艰巨。我们坚信有省委、省政府和市委的正确领导，有全市人民的大力支持，有战危机、抗灾害的宝贵经验，有后续项目的保障支撑，只要我们坚定信心，抢抓机遇，勇于进取，敢于争先，就一定能经受新考验，再创新辉煌!

做好今年政府工作，必须以党的十七大和十七届三中、四中全会精神为指导，用科学发展观统领经济社会发展全局，认真贯彻落实省委经济工作会议、省“两会”和市委经济工作会议精神，按照重在持续、重在提升、重在统筹、重在为民的要求，围绕转变经济发展方式，着力扩大有效需求，着力调整经济结构，着力统筹城乡协调发展，着力推进改革开放，着力改善民生与促进社会和谐，全面完成“十一五”规划目标任务，促进全市经济社会又好又快发展。

综合考虑各方面的因素，2010年我市经济社会发展的主要预期目标和约束指标是：生产总值增长12%左右;地方财政一般预算收入增长10%;全社会固定资产投资增长22%;社会消费品零售总额增长17%;外贸出口增长11%;实际利用外商直接投资增长15%;城镇居民人均可支配收入增长8%，农民人均纯收入增长9%;人口出生率、城镇登记失业率、单位生产总值能耗、化学需氧量和二氧化硫排放量控制在省定目标以内。

需要说明的是，确定今年生产总值增长12%左右的目标，主要是基于我市的实际，基于对当前形势和今后走势的判断把握，考虑了保持经济平稳较快增长的需要，考虑了扩大就业、增加收入、改善民生的需要，考虑了增强经济增长潜力、提高区域竞争力的需要。这样安排有利于把经济社会发展的着力点引导到推动结构调整和产业升级上来，引导到促进经济社会全面协调与可持续发展上来，引导到解决群众生产生活中的实际问题上来。

围绕实现上述目标，今年政府工作的首要任务是，既要保持经济平稳较快发展，又要调结构、促转型，惠民生、促和谐，在转变发展方式上取得突破，在多办好事实事上持续作为。做好今年的政府工作，要着重把握好以下原则：一是坚持发展是第一要务，努力保持经济平稳较快增长，避免大起大落，切实把经济社会的发展纳入科学发展的轨道，在工作中把握方向、掌握节奏，产业升级、增强后劲，夯实基础、强化保障，关注民生、促进和谐，做到速度和质量相统一、结构和效益相协调，为长期持续较快发展提供支撑。二是坚持以扩大需求为主导，在突出投资对经济拉动的同时，更加注重扩大内需特别是消费需求对经济增长的拉动力，挖掘消费潜力，放手发展现代服务业，扩充总量，壮大实力，优化结构，提高质量。三是坚持以结构调整为重点，把推动自主创新与改造提升传统产业、培育战略性新兴产业结合起来，积极运用高新技术、先进适用技术改造提升传统产业，着力构建现代产业体系，培育新的经济增长点，全面提升产业竞争力，巩固和壮大发展基础。四是坚持以推进城镇化为依托，把加快新型工业化、城镇化步伐作为解决“三农”问题的根本途径，推进生产要素合理流动和优化配置，加快城乡基本公共服务均等化，以工促农，以城带乡，统筹城乡协调发展。五是坚持以改革开放为动力，弘扬敢闯敢试的精神，培育创新创业的激情，优化招商引资的环境，通过改革增强发展活力，通过开放提升发展速度。六是坚持以改善民生为目的，按照公共财政阳光向公益事业普照、收入分配向全体公民倾斜、改善民生举措向弱势群体惠及的要求，坚持不懈地为老百姓多办看得见、摸得着、享得了的好事实事。

三、2010年主要工作任务

今年要重点抓好以下八个方面的工作：

(一)以服务企业为核心，加强经济运行调节。

一是继续深化企业服务年活动。按照规模以上工业增加值增速达到15%，企业利税突破150亿元，亿元企业突破150家的发展目标，把为企业服务的重点转向促进企业转型升级、加快企业战略重组、培育壮大市场主体上来。筛选100户工业企业作为重点服务企业，继续落实领导联系企业制度，加快推进新建项目的投产工作，尽早形成新的经济增长点。加强产销衔接，帮助企业开拓市场。全面落实促进中小企业发展的政策措施，推进中小企业健康发展。继续实施品牌战略和质量兴市战略，抓好产品质量、工程质量和服务质量，提高企业质量效益。

二是强化投资服务与管理。优化投资结构，扩大民间投资，完善投资促进机制，进一步落实重点项目协调推进机制和重大项目联审联批机制，简化办事程序，提高审批效率，服务项目早投产早见效，保持投资对经济增长的拉动力。

三是努力扩大消费需求。落实家电下乡、汽车摩托车下乡、汽车以旧换新等政策，“万村千乡市场工程”要覆盖全市85%以上的行政村。培育文化、旅游、信息、家政、养老、健身、培训等新的消费热点，发展连锁经营、特许经营、电子商务、物流配送等现代流通业和网上购物、网络教育等新型消费方式。加强住房保障制度建设，完善促进住房消费的政策，促进房地产市场健康发展。

四是做好生产要素保障。优化信贷结构，加强对重点领域和薄弱环节的信贷支持。畅通银政企沟通渠道，促进银企合作，加大对企业的资金支持力度。做好土地盘活挖潜和收购储备工作，加强煤电油气运供应的协调，提高保障能力。加大对市场价格的监管力度，确保物价保持在合理水平。落实税费优惠政策，进一步减轻企业和居民负担。

(二)以产业集聚区为载体，促进结构调整产业升级。

一是加快产业集聚区建设。坚持产城互动、融合发展，提高产业集聚区的承载力、吸引力和竞争力。按照企业集中布局、产业集群发展、资源集约利用、功能集合构建的原则，科学编制并实施产业集聚区规划。进一步增强产业集聚区建设的投资强度，重点加强基础设施、入驻项目、投融资平台、管理效能和标准化厂房建设。落实好城乡建设用地增减挂钩、异地投资税收分享和有关税收实行“核定基数、超收返还”优惠政策。严格奖惩措施，按照产业集聚区建设考核办法，坚决兑现奖惩。

二是切实转变发展方式。围绕煤及煤化工、铝及铝深加工、农副产品深加工、机械制造等主导产业，积极构建现代产业体系;围绕碳纤维、太阳能、生物制药等新材料、新能源、新工艺，加快引进和培育发展潜力大、带动作用强的新兴产业。推进煤电、煤层气开发和电源项目建设，打造商丘能源基地。加快电解铝、铝合金、铝型材、铝板带、铝箔坯料、轮毂等项目建设，打造商丘铝深加工基地。进一步拉长面及面制品加工、畜牧养殖及肉奶加工、食用菌、林果及加工、林板浆纸一体化、棉纺业产业链条，打造商丘农副产品深加工基地。加快醋酸、乙二醇、醇醚等煤化工高端项目建设及石油化工项目的谋划进度，打造商丘化工基地。推进企业战略重组，开展产业链整合，上下游联合，扩大企业战略协作。大力发展现代服务业，加大旅游投入和宣传推介力度，完善旅游管理体制，培育和发展文化、旅游产业，大力发展古色游、红色游、绿色游、特色游等旅游业态。加快发展金融服务业，积极引进股份制银行、证券公司、保险公司等机构。升级改造商丘农产品中心批发市场、中原车城等专业市场，做好文化专业市场规划建设，繁荣光彩大市场，加快商丘区域性商贸物流中心城市建设步伐。

三是大力推进自主创新。进一步加大对科技的投入力度，加强科技人才的培养引进，支持企业孵化器、研发中心建设，培育壮大自主创新主体。围绕主导产业，开展重大科技专项攻关活动，保护知识产权。创新政府资金支持机制，拓宽科技成果转化为现实生产力的渠道。

四是扎实开展节能减排。建立和完善节能减排统计指标体系、监测体系和考核体系，推进建筑、交通、公共机构等重点领域节能工作，加大淘汰落后产能的力度，大力发展循环经济，推进工业废渣、废气、废水等废弃资源的再生利用，促进可持续发展。继续加强重点流域、重点区域、重点行业污染的综合整治。加强饮用水水源保护，健全水环境预警制度和水环境生态补偿机制，确保人民群众的饮水安全。

(三)以发展现代农业为目标，夯实“三农”工作基础。

一是巩固提高粮食综合生产能力。按照稳定面积、主攻单产、增加总产、提高效益的要求，实施粮食生产发展规划，抓好粮食生产核心区建设，确保商丘粮食综合生产能力稳定在120亿斤左右。加强农田水利基本建设，改善农业生产条件，加快5座病险水库除险加固工程和清淤扩容进度，抓好引黄灌区配套工程建设，深入实施“井井通电”工程，完成中低产田改造11万亩。

二是深入推进农业结构调整。坚持用工业的理念发展农业，按照科技武装、规模经营、拉长链条、提高效益的要求，大力发展农副产品加工业，培育壮大农业产业化龙头企业。今年省级以上龙头企业发展到50家，规模化种植小区和标准化养殖小区分别发展到100个和500个。积极引导发展多种形式的农民专业合作组织，培育优质农产品品牌。加快县级农产品质量检测体系建设，完善基层动物防疫网络。推进林业生态市建设，发展林业产业、林下种养殖经济和花卉产业，实现改善生态环境和经济效益双丰收。

三是认真落实强农惠农政策。不折不扣地把粮食直补、综合直补、良种补贴、农机具购置补贴、农资综合直补及时发放到农民手中，严格执行粮食最低保护价收购政策，建立完善保护种粮农民利益的长效机制，调动农民群众种粮的积极性。

四是加大农村劳动力的培训转移力度。积极拓展农民就业渠道，引导农民有序外出就业，鼓励农民就地就近转移就业，扶持农民工返乡创业。加强技术培训，提高农民的劳动技能和自主创业能力;大力发展劳务经济，增加农民收入。

(四)以招商引资为重点，深入推进改革开放。

一是加大重点项目建设力度。实施“3323工程”，即围绕“三大跨越”的目标，在农业规模发展、工业转型升级、节能减排和城乡一体化建设方面实施300个项目，总投资超过2000亿元，当年完成投资300亿元以上。建立项目谋划、联审联批、领导联系重大项目、项目建设进度推进、考核奖惩五项工作机制，全力服务重点项目建设。2010年全市第一批初步安排重点建设项目228个，总投资1112亿元，当年计划投资270亿元。加快永煤集团龙宇煤化工基地二期、神火集团1�60万千瓦电厂、2�30万千瓦热电联产机组、庄周500KV输变电站二期扩容、永城500KV输变电站、阳光铝材年产15万吨铝板带项目、郑煤集团商丘中亚化工公司12万吨醇醚项目、河南煤化集团千吨级碳纤维项目、科迪集团年产50万吨综合面业等在建项目建设进度，尽快形成新的经济增长点。全力做好中石油商丘千万吨炼油项目纳入国家“十二五”规划的前期工作。

二是深入开展大招商活动。利用产业转移的难得机遇，全方位开展招商活动。创新招商方式，突出请进来招商，让客商到商丘亲身感受投资环境和发展商机;突出产业集聚区招商，围绕主导产业，努力承接转移;突出专业对口小分队招商，做到点对点、一盯一，常抓不懈;突出以商招商，发挥企业主体招商作用，做到引来一个，发展一片;突出产业链招商，依托现有企业，延长产业链条，发展下游产品和高端产品。探索委托招商，引入市场化招商机制，借助中介机构、行业组织，利用他们的专业知识、专业队伍以及所拥有的资源招商。组织好重大招商活动，注重实际效果，提高工作水平。积极推进外来投资项目无偿代理制和项目联审联批制，加快项目落地速度。强化招商项目的跟踪问效，完善分级负责制，建立招商引资评价奖惩机制，严格落实招商引资奖惩办法。继续坚持千名优秀干部离岗招商制度。积极参与第六届中国河南国际投资贸易洽谈会、世界华商高峰论坛、河南～东盟投资贸易洽谈会。办好2010国际华商文化节、第六届面品博览会。

三是深化重点领域改革。继续深化国有企业改革，着力引进有实力的战略投资者，完善企业法人治理结构，转换企业经营机制。继续推进商贸、粮食企业改革改制，完善国有资产监管和运营体系，将现国有企业与原来的行业管理部门脱钩，实行统一管理，增强企业活力。完成县级政府机构改革，开展大科室试点工作。深化医药卫生体制改革，完善基本医疗保障制度，启动实施国家基本药物制度，不断完善新农合医疗保障体系，全面实现医疗救助与新农合、城镇职工和居民医保同步结算。深化文化体制改革，启动国有文艺院团转企改制，完成经营性文化事业单位、县以上国有电影发行放映单位转企改制任务，加快电台、电视台制播分离改革步伐。在依法自愿有偿流转的基础上，发展适度规模经营，开展以土地承包经营权置换社会保障、以宅基地置换城镇房产试点工作。大力发展农民专业合作社，提高农业生产经营组织化程度。加快农村金融体制创新，支持发展村镇银行、贷款公司、农村资金互助社等农村金融机构，让更多的生产要素流向农村，支持“三农”，促进农村经济发展。深化林权制度改革。推进警务机制改革。

四是积极实施口岸大通关建设，加强与上海、连云港等城市的海关协作，建设电子口岸和铁路口岸，实施商丘大通关，提高进出口贸易便利化水平。大力发展民营经济，认真贯彻落实支持民营经济发展的政策措施，切实维护民营企业的合法权益，真正使民营经济成为最有活力的经济增长极。

(五)以城镇建设为依托，加快城乡统筹发展步伐。

一是修编完善现代城镇体系规划。完成《商丘城市发展战略规划》，提高规划区内控制性详细规划和专业规划覆盖率。加快商丘新区规划编制的步伐，提高商丘新区的规划编制水平。编制商虞一体化发展战略规划，加快对接步伐。完成中心城区生态水系、综合交通体系和旧城改造规划，编制火车站、火车南站周边地区控制性详细规划。严格城市规划和用地管理，认真解决批而未征征而未供土地问题，深入开展违法违规用地建设集中整治活动。

二是着力打造综合交通体系。加快商周高速公路二期工程、连霍高速商丘段改扩建工程建设进度;开工建设郑徐客运专线商丘段、商丘机场、济祁高速公路商丘永城段和邢商永地方铁路一期工程邢口至商丘段，争取将沱浍河航运开发项目列入国家“十二五”规划，积极做好310国道和105国道升级改造的前期工作，加快农村公路建设，实施危桥改造，构建“乡乡联、县县通”农村公路网络。对商丘市综合交通体系开展科学规划，努力把商丘打造成为集铁路、公路、民航、内河航运、管道运输及电信传输为一体的综合交通枢纽城市。

三是加强城市基础设施建设。巩固创建国家园林城成果，加快旧城改造和“城中村”改造步伐，加快实施道路、管网、亮化、绿化工程，建成10万吨污水处理厂，抓好配套管网工程。加快西气东输二线工程建设进度。大力实施弱电管网入地工程建设。全面完成110条背街小巷综合整治任务。积极推进应天公园、梅园、翠竹园、月季公园建设。投资1.2亿元资金，启动城区水系污水管网规划建设工作。理顺水管体制，加大污水处理费和水资源费征收力度。对运河西段、中段北岸和东段南岸实施综合治理。努力完成房地产投资20亿元，开工面积90万平方米。适时启动国家卫生城、省级文明城创建工作。

四是抓好县城和小城镇建设。把县城建设作为加快县域经济发展的重要载体，推动特色产业、优势项目向县城集聚，壮大县域经济，增强人口转移承载力。围绕建设常住人口20万人宜居城市的目标，大力加强道路、绿化、管网、垃圾处理、电力、通讯等基础设施建设，完善县城功能。坚持因地制宜、产业兴镇的原则，建成一批特色明显、实力较强的中心乡镇，增强辐射带动能力。破除户籍壁垒，凡是进城农民有稳定居所和稳定经济来源，就可以进城落户。

五是扎实推进新农村建设。结合乡镇土地利用总体规划修编，完成县(市)域村镇体系规划、中心村规划和所有乡镇的规划修编任务。抓好32个农村土地综合整治试点村建设，3年内完成全市农村土地综合整治“百村”工程。加大涉农资金项目的整合力度，不断向新农村建设倾斜，鼓励社会各界积极参与新农村建设。继续推进“百村示范”工程，认真落实示范村建设与扶贫开发整村推进和驻村帮扶“三结合”工作机制。每个县(市、区)重点抓好2个省级和10个市级示范村建设。健全完善乡镇生活垃圾收集压缩中转运输系统，深入开展“清洁家园”行动，搞好村容村貌整治。

(六)以社会进步为方向，不断增强人民群众的幸福感。

一是大力实施积极就业政策。把就业作为民生之首，大力实施“就业促进行动计划”。开发公益性就业岗位，做好“零就业家庭”、“4050”人员等就业困难群众的就业援助。加强劳动者技能素质培训，重点解决城镇零就业家庭人员的就业援助工作。积极构建和谐劳动关系，依法保障劳资双方合法权益。深入开展全民创业活动，以创业促进就业、以就业促进发展、以发展促进和谐。

二是积极促进教育均衡发展。合理配置义务教育办学资源，提高义务教育阶段教育教学水平。加大城区中小学布局调整，进一步解决城区中小学入学难问题。继续做好“两免一补”工作，逐步提高城区中小学公用经费标准。推进职教攻坚计划，创新职业教育体制机制，引导和推动职业教育集团化办学。加强学科建设，形成办学特色，提高高等教育发展水平。认真做好职教强县和全省示范性职业学校创建工作。做好商丘职业技术学院通过国家验收和商丘科技职业学院专升本工作。

三是提升公共卫生服务能力。继续做好甲型H1N1流感和手足口病防控工作，完善社区卫生服务体系，提高新型农村合作医疗保障水平。全面加强人口和计划生育工作，稳定低生育水平，提高出生人口素质。做好第六次人口普查工作。深入开展食品药品安全专项整治，严格落实市场准入、产品质量追溯和召回制度，保障公众食品药品安全。

四是繁荣城乡文化生活。开展“舞台艺术送农民”、文化下乡、电影下乡活动。加快市县两级公共图书馆、文化馆、博物馆建设。继续实施乡镇文化站建设工程、文化信息资源共享工程、农家书屋工程。扶持民权王公庄画虎产业发展，打造商丘文化产业标志性品牌。

五是完善社会保障体系。建立完善社会保险制度，扩大社保覆盖面，逐步解决特殊困难群体的参保问题。抓好梁园区、虞城县全国新型农村社会养老保险试点。健全城乡医疗救助制度、孤儿救助、艾滋病致孤儿童救助机制，提高机构收养孤老、孤儿、孤残人员的供养标准和城镇基本医疗保险待遇。加强对困难群众的社会救助，建立临时社会救助制度。完善最低生活保障制度，保障和改善困难群众的基本生活。

六是维护社会和谐稳定。围绕社会矛盾化解、社会管理创新、公正廉洁执法总体要求，进一步健全和落实信访维稳工作责任制，着力构建“大排查”、“大调解”、“大接访”工作格局，坚持完善领导干部接访制度，把问题在第一时间解决，把矛盾在第一现场化解。加强应急管理工作，构建群防群治突发事件管理机制，提高全市各级政府应对突发公共事件的能力。加强社会治安综合治理，健全社会治安防控体系，依法严厉打击各类犯罪活动，提升群众安全感。进一步落实安全生产责任制，建立市县乡三级安全生产监控网络，及时排查消除安全隐患，坚决遏制重特大安全事故的发生。加强民族团结，促进宗教和谐，营造共同进步的良好社会氛围。

(七)以改善民生为目的，让人民群众得到更多实惠。

今年继续办好关系人民群众切身利益的十件实事：

一是提高社会保障水平。为全市8.5万名企业退休人员增加基本养老金，人均在原每月1020元的基础上月均增资147元;市直城镇职工基本医疗保险最高支付限额提高到6万元，大额补充医疗保险最高支付限额提高到29万元，在一个自然年度内最高支付限额提高到35万元。新型农村合作医疗筹资水平由2009年每人每年100元提高到150元，大额医疗费用支付限额提高到6万元;提高农村五保对象集中供养标准，农村五保对象集中供养标准由1600元提高到不低于2000元，分散供养标准由1100元提高到不低于1200元。

二是为低收入群体提供住房保障。今年要建成廉租房8500套、经济适用房5000套，完成棚户区改造25万平方米。

三是对“4050”人员就业实施“六帮扶、一托底”，即开展素质提升、岗位对接、择业信息、自主创业、维护权益、“一对一”针对性帮扶活动，政府对其托底安置。今年政府购买公益性岗位5000个，帮助“4050”人员实现再就业，动态消除零就业家庭。

四是免费和补贴培训农民工。实施“阳光工程”、“雨露计划”，开展农村劳动力职业技能培训，免费培训、补贴培训8万人次以上。

五是优化市民居住生活环境。完成城区110条背街小巷整修工程，方便居民出行;开展梁园区早期人防工程安全隐患治理，年内消除隐患面积1200平方米;建设城区垃圾公厕联体中转站15座、新建水冲公厕15座、改造旱厕40座;在城区公交车停靠点设置垃圾箱326个。综合整治城市内河水系，完成《商丘市区生态水系规划》编制，对包河、忠民沟、万堤沟、蔡河实施综合治理，完成民主东路包河桥改造工程。

六是改善城区交通状况。大修改造建设路，升级改造神火大道(北海路～火车站)、南京路(平原路～火车南站)、凯旋路(北海路～310国道)、文化路(睢阳路～金桥路)未改造路段，综合整治平原路、归德路、民主路，新修长江西路。综合治理火车站商业区域。

七是关心救助弱势群体。开工建设商丘市智障、精神病及重度残疾人托养中心。加强法律援助，扩大援助范围，使符合条件的困难群众都能得到及时无偿的法律帮助，实现应援尽援。

八是改善农村居民生产生活条件。解决30万人安全饮水问题;修建农村公路350公里;加大农村环境治理力度，建设115个乡镇生活垃圾中转站。

九是改扩建3所城区中小学校。扩建市实验中学初中部，改造文化路小学，实施特殊教育学校搬迁新建，改善城区办学条件。

十是提高公共安全保障能力。实施“报警编码标识”系统建设工程，在城区范围内对电线杆、路灯杆进行统一编号，方便群众求助和报警;在城区主要道路、城市出入口、学校等重要位置、重要部位安装监控设备，促进城区公共安全;加强防震减灾工作，在城市重点区域内建设2处应急避难场所，提高应对重大突发灾害的能力;推进农村十户联防，扩大农村技防设施入户覆盖率，增强农民群众安全感。

(八)以求实求效为原则，切实加强政府自身建设。

一是突出发展主题。当前我们面临着难得的发展机遇，肩负着推动发展的历史重任，我们一定要抓住机遇，乘势而上，千方百计地用足政策用活政策，千方百计地争取项目大上项目，千方百计地引进资金引进人才，千方百计地鼓励创新支持创业，以只争朝夕、奋发有为的精神状态，渡危机促发展，既保持当前的经济增长态势，又为商丘今后的长远发展打牢基础、备足后劲。

二是强化宗旨意识。以人为本、执政为民是党组织的要求，是人民政府的职责。每一个政府公务人员都要始终牢记全心全意为人民服务的宗旨，学会换位思考，真诚倾听群众呼声，真实反映群众愿望，真情关心群众疾苦，站在老百姓立场上看问题、办事情、做决策。多做雪中送炭工作，解决好群众最关心、反映最强烈的问题，真正让人民群众得到实惠，真正让惠民措施长期坚持，形成制度，落到实处。

三是提高服务能力。新形势新任务，对政府工作也提出了新的更高要求。要进一步强化政府服务的理念，切实转变机关工作作风，腾出更多的时间和精力投入到促增长、促转型上来。深入开展“两转两提”活动，发挥行政效能电子监察系统的作用，提升行政服务大厅的服务水平和整合能力，打造一流的综合行政服务机构。积极推进政务公开，发挥“12345”市长热线、市长信箱和群众来访办理等沟通平台的作用，努力打造阳光政府。进一步强化政府目标管理，着力提高行政效率和工作质量。

四是务求工作实效。经济社会发展遇到的困难越多，越要弘扬求真务实精神，强化实干意识，形成求实作风。我们要察实情，深入基层、深入群众，问政于民、问需于民、问计于民，提高决策水平;我们要干实事，多做打基础、管长远、利全局的工作，多为老百姓做好事、办实事、解难事，努力创造出经得起历史、实践和人民检验的政绩;我们要重实效，不说空话，不放空炮，少提口号，多干实事，说到做到，说好做好，不做表面文章，不搞形式主义。

五是坚持从严治政。切实把政府工作纳入依法运行的轨道，严格执行人大及其常委会决议、决定，自觉接受人大及其常委会的法律监督，主动接受政协的民主监督，认真办理人大代表建议、议案和政协委员提案。严格落实廉政建设责任制，坚持用制度管权、管人、管事，确保权力规范运行、透明运行、阳光运行。政府及其工作人员都要勤勉尽责，秉公用权，堂堂正正做人，踏踏实实做事，干干净净做官，争当人民满意的公务员，建设廉洁高效政府。

各位代表，新的一年，困难和挑战考验着我们，责任和使命激励着我们。让我们紧密团结在以胡锦涛同志为总书记的党中央周围，高举中国特色社会主义伟大旗帜，以邓小平理论和“三个代表”重要思想为指导，深入贯彻落实科学发展观，在省委、省政府和市委的正确领导下，坚定信心，抢抓机遇，迎难而上，埋头苦干，为建设全市人民满意的幸福家园而努力奋斗!
